# Supplementary material for: Altered microRNA Transcriptome in Cultured Human Liver Cells upon Infection with Ebola Virus
Source: Int J Mol Sci. 2021 Apr 6;22(7):3792. doi: 10.3390/ijms22073792 (PMC8038836; doi:10.3390/ijms22073792)
Supplement: Supplementary file 1 [file ijms-22-03792-s001.zip › Supplementary_File/C_ GO_Analysis_Results/16-30nt_go_Makona-96h-Huh7_vs_Control-96h-Huh7_up.mature_mirna_targets/BP_Pvalue_tree.pdf]

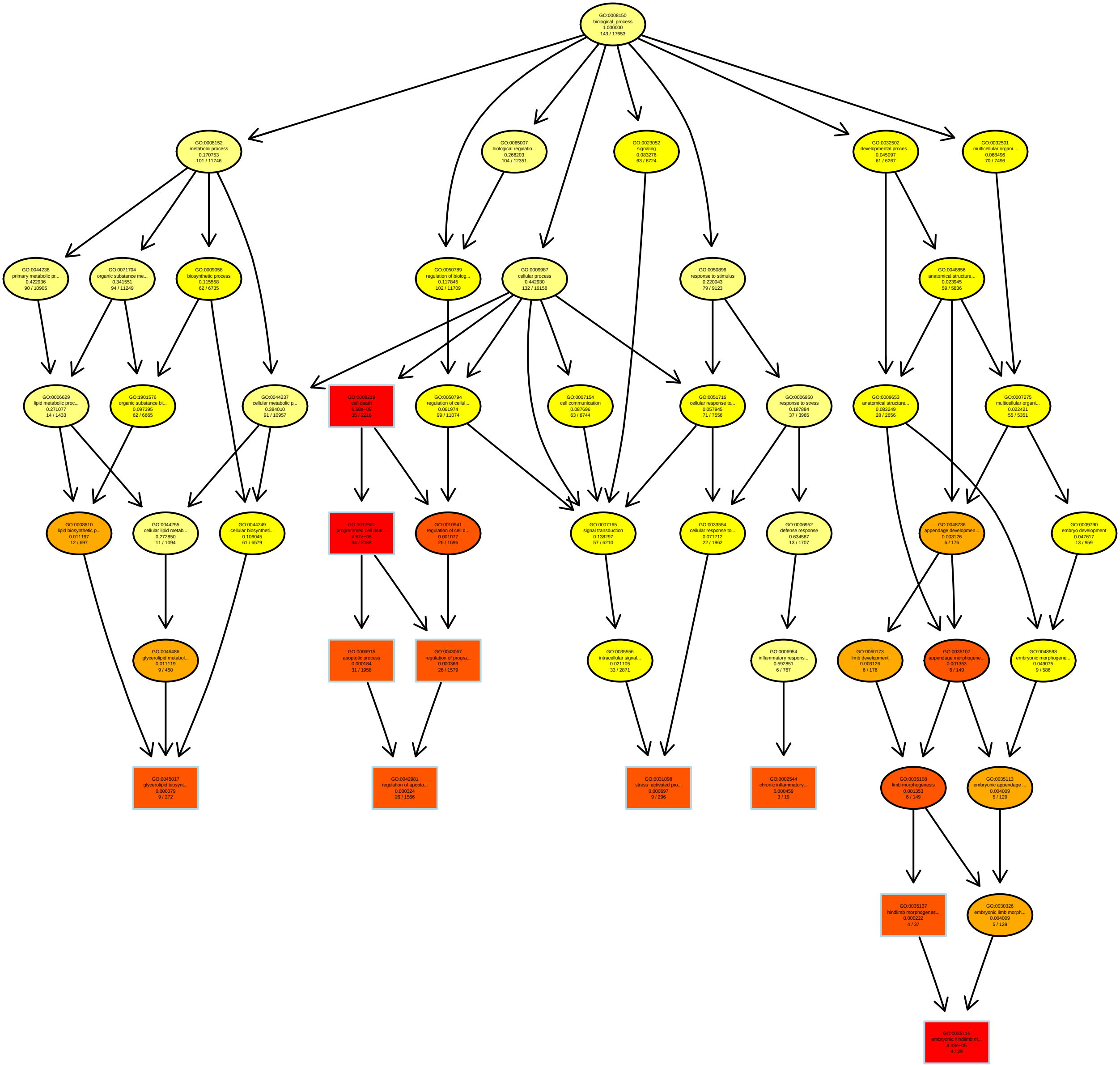

GO:0008150 biological_process 1.000000 143 / 17653
GO:0008152 metabolic process 0.170753 101 / 11746
GO:0065007 biological regulatio... 0.266203 104 / 12351
GO:0023052 signaling 0.083276 63 / 6724
GO:0032502 developmental proces... 0.045097 61 / 6267
GO:0032501 multicellular organi... 0.068496 70 / 7496
GO:0044238 primary metabolic pr... 0.422936 90 / 10905
GO:0071704 organic substance me... 0.341551 94 / 11249
GO:0009058 biosynthetic process 0.115558 62 / 6735
GO:0050789 regulation of biolog... 0.117845 102 / 11709
GO:0009987 cellular process 0.442930 132 / 16158
GO:0050896 response to stimulus 0.220043 79 / 9123
GO:0048856 anatomical structure... 0.023945 59 / 5836
GO:0006629 lipid metabolic proc... 0.271077 14 / 1433
GO:1901576 organic substance bi... 0.097395 62 / 6665
GO:0044237 cellular metabolic p... 0.384010 91 / 10957
GO:0008219 cell death 6.58e−05 35 / 2216
GO:0050794 regulation of cellul... 0.061974 99 / 11074
GO:0007154 cell communication 0.087696 63 / 6744
GO:0051716 cellular response to... 0.057945 71 / 7556
GO:0006950 response to stress 0.187884 37 / 3965
GO:0009653 anatomical structure... 0.083249 28 / 2656
GO:0007275 multicellular organi... 0.022421 55 / 5351
GO:0008610 lipid biosynthetic p... 0.011197 12 / 697
GO:0044255 cellular lipid metab... 0.272850 11 / 1094
GO:0044249 cellular biosyntheti... 0.106045 61 / 6579
GO:0012501 programmed cell deat... 4.67e−05 34 / 2088
GO:0010941 regulation of cell d... 0.001077 26 / 1696
GO:0007165 signal transduction 0.138297 57 / 6210
GO:0033554 cellular response to... 0.071712 22 / 1962
GO:0006952 defense response 0.634587 13 / 1707
GO:0048736 appendage developmen... 0.003126 6 / 176
GO:0009790 embryo development 0.047617 13 / 959
GO:0046486 glycerolipid metabol... 0.011119 9 / 450
GO:0006915 apoptotic process 0.000184 31 / 1958
GO:0043067 regulation of progra... 0.000369 26 / 1579
GO:0035556 intracellular signal... 0.021105 33 / 2871
GO:0006954 inflammatory respons... 0.592851 6 / 767
GO:0060173 limb development 0.003126 6 / 176
GO:0035107 appendage morphogene... 0.001353 6 / 149
GO:0048598 embryonic morphogene... 0.049075 9 / 586
GO:0045017 glycerolipid biosynt... 0.000379 9 / 272
GO:0042981 regulation of apopto... 0.000324 26 / 1566
GO:0031098 stress−activated pro... 0.000697 9 / 296
GO:0002544 chronic inflammatory... 0.000459 3 / 19
GO:0035108 limb morphogenesis 0.001353 6 / 149
GO:0035113 embryonic appendage ... 0.004009 5 / 129
GO:0035137 hindlimb morphogenes... 0.000222 4 / 37
GO:0030326 embryonic limb morph... 0.004009 5 / 129
GO:0035116 embryonic hindlimb m... 8.38e−05 4 / 29
